# Antineoplastic Activity of Pazopanib in Anaplastic Thyroid Cancer in Primary Culture

**DOI:** 10.3390/ijms24032398

**Published:** 2023-01-25

**Authors:** Silvia Martina Ferrari, Giusy Elia, Francesca Ragusa, Sabrina Rosaria Paparo, Valeria Mazzi, Armando Patrizio, Simona Piaggi, Enke Baldini, Marco Centanni, Concettina La Motta, Alessandro Antonelli, Poupak Fallahi

**Affiliations:** 1 Department of Clinical and Experimental Medicine, University of Pisa, 56126 Pisa, Italy; 2 Department of Surgery, Medical and Molecular Pathology and of Critical Area, University of Pisa, 56126 Pisa, Italy; 3 Department of Translational Research and New Technologies in Medicine and Surgery, University of Pisa, 56126 Pisa, Italy; 4 Department of Emergency Medicine, Azienda Ospedaliero-Universitaria Pisana, 56126 Pisa, Italy; 5 Department of Surgical Sciences, Sapienza University of Rome, 00161 Rome, Italy; 6 Department of Medico-Surgical Sciences and Biotechnologies, Sapienza University of Rome, 00185 Latina, Italy; 7 Endocrine Unit, AUSL Latina, 04100 Latina, Italy; 8 Department of Pharmacy, University of Pisa, 56126 Pisa, Italy

**Keywords:** pazopanib, anaplastic thyroid cancer, primary cell cultures, tyrosine kinase inhibitors, proliferation, apoptosis, migration, invasion

## Abstract

Anaplastic thyroid cancer (ATC) is a rare and rapidly fatal human cancer. Its usual treatment includes the combination of surgery, external hyperfractionated radiation therapy, and chemotherapy. These treatments permit achieving about 6–10 months of median survival. For this reason, it is challenging to predict the ATC patient clinical therapy responsiveness. Pazopanib is a multitarget tyrosine kinase inhibitor of VEGF receptors, PDGF, and c-Kit. Until now, the effect of pazopanib in primary human ATC cells (pATC) has not been reported in the literature. The aim of our study was to evaluate in vitro the antineoplastic effect of pazopanib in pATC. Surgical thyroidal tissues were collected from five patients with ATC, from thyroid biopsy at the moment of first surgical operation. An inhibition of proliferation, migration, and invasion, and an increase in apoptosis were demonstrated upon treating pATC cells with pazopanib (*p* < 0.05). Moreover, pazopanib was able to significantly decrease the VEGF expression in pATC cells (*p* < 0.05). To conclude, in this study, we demonstrate the antineoplastic activity of the antiangiogenic inhibitor, pazopanib, in human pATC in vitro.

## 1. Introduction

Thyroid cancer (TC) is the most frequent endocrine tumor all over the world, with a growing incidence in the last years [1,2]. Thyroid follicular epithelial-derived carcinomas, called differentiated thyroid carcinomas (DTCs), represent more than 90% of TCs and are subdivided into papillary TCs (PTCs; ~90%), follicular TCs (FTCs; ~10%), and anaplastic TCs (ATCs, <2%) [1,2,3]. 

ATC is a rare and rapidly fatal human cancer [1,4]. Its usual treatment includes the combination of surgery [5], external hyperfractionated radiation therapy, and chemotherapy, with doxorubicin and cisplatin. These treatments permit achieving about 6–10 months of median survival [4].

For this reason, it is challenging to predict the ATC patient clinical therapy responsiveness, and it may be helpful to identify a valid systemic treatment to improve the quality of life of these patients [6,7]. 

The new advancements in the understanding of the molecular pathways at the basis of ATC development have led to the discovery of new drugs. 

Recently, small-molecule inhibitors of tyrosine kinase (TKI) pathways involved in the progression of ATC have been developed. These TKIs act against aggressive and refractory TCs, and they have attained the approvement of regulatory agencies from the US (FDA) and EU (EMA). These TKIs target the intracellular TK associated with various receptors (i.e., vascular endothelial growth factor receptor (VEGFR)2/3, fibroblast growth factor receptors (FGFRs), platelet-derived growth factor receptor (PDGFR), rearranged during transfection (RET), KIT, epidermal growth factor receptor (EGFR), Tie2, and c-Met) [8,9,10].

Recently, the US FDA approved sorafenib [11], lenvatinib [12], and cabozantinib [13] for the therapy of recurrent or metastatic, radioactive iodine refractory DTC (RAIR-DTC), as well as cabozantinib [14] and vandetanib [15,16] for MTC. Moreover, the combination of dabrafenib and trametinib received approval for ATC with ^V600E^BRAF mutation [17,18]. 

We demonstrated that vandetanib and lenvatinib have an important antitumoral effect in vitro in primary human ATC cell cultures (pATC), as well as in xenotrasplants of ATC in nude mice in vivo [19,20]. The antineoplastic action of vandetanib and lenvatinib was also demonstrated in pATC cells established from biopsy, such as from fine needle aspiration (FNA) cytology [21]. 

Moreover, we showed the antitumoral and antiangiogenic activity of other compounds, such as pyrazolo [3,4-d]pyrimidine (with antiangiogenic action and ability to inhibit VEGFR, EGFR, and the RET TK) CLM3 in pATC cells [22]. The antiangiogenic cyclic amide CLM94 had an antitumor effect in vitro and in vivo in pATC [23], and the pyrazolo[3,4-d]pyrimidine CLM29 and CLM24 compounds exhibited a strong antitumor activity in pATC cells and in the continuous 8305C cell line [24].

Predictive values (PVs) for clinical benefit can be achieved through disease-oriented in vitro drug screening in human cancer cell lines [25,26], since a 60% positive PV and a 90% negative PV have been shown [27]. For this reason, in vitro drug testing could help clinicians in the choice of the right, therapy avoiding the administration of inactive compounds.

Thyroid continuous cell lines have been used as preclinical models, but these cells adapt to in vitro growth conditions, thus losing the intrinsic features of the primary tumor. Therefore, in order to hamper these important limitations, primary human cell cultures have been set up as monolayer cultures, and their biological behavior has been evaluated.

Moreover, as reported above, primary TC cells can be established from surgical or FNA cytology samples from DTC or ATC. For these reasons, a personalization of the therapy could be allowed through the possibility to test the chemosensitivity of pATC from each subject to several drugs, thereby increasing the efficacy of the treatment [28].

Pazopanib (Votrient) is a multitarget TKI of VEGF receptors, PDGF, and c-Kit, approved by the FDA for the treatment of advanced renal cell carcinoma and advanced soft-tissue sarcoma. This antiangiogenic drug showed promising clinical activity in DTC patients, and it was concluded that pazopanib could represent a promising therapeutic option for these patients [29,30,31]. Moreover, the antineoplastic effect of pazopanib has also been reported in in vitro studies in ATC [29,32,33].

The aim of this study was to evaluate the antineoplastic effect of pazopanib in primary ATC cells.

## 2. Results

**Figure 1:**
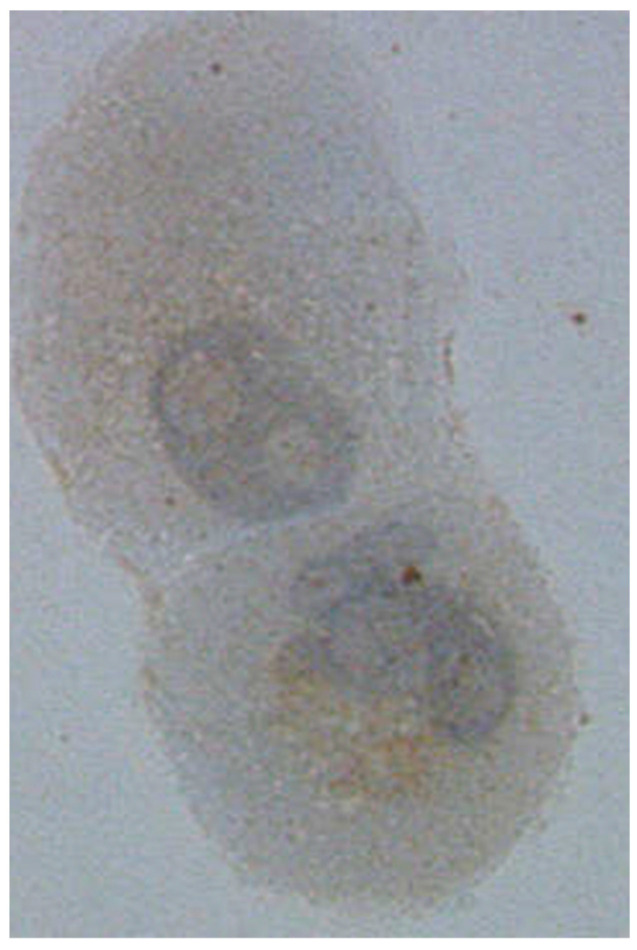
Anaplastic thyroid cancer cells cultured from surgical thyroidal samples stained with cytokeratin (magnification, 400×).

The lack of expression of thyroglobulin (Tg), thyroid-stimulating hormone (TSH) receptor, thyroperoxidase (TPO), and sodium/iodide symporter (NIS) was shown by immunohistochemistry. A partial and focal positivity to cytokeratin was revealed by immunocytochemistry (Figure 1). DNA fingerprinting reported a pattern identical to that of the original tumoral tissue.

**Figure 2:**
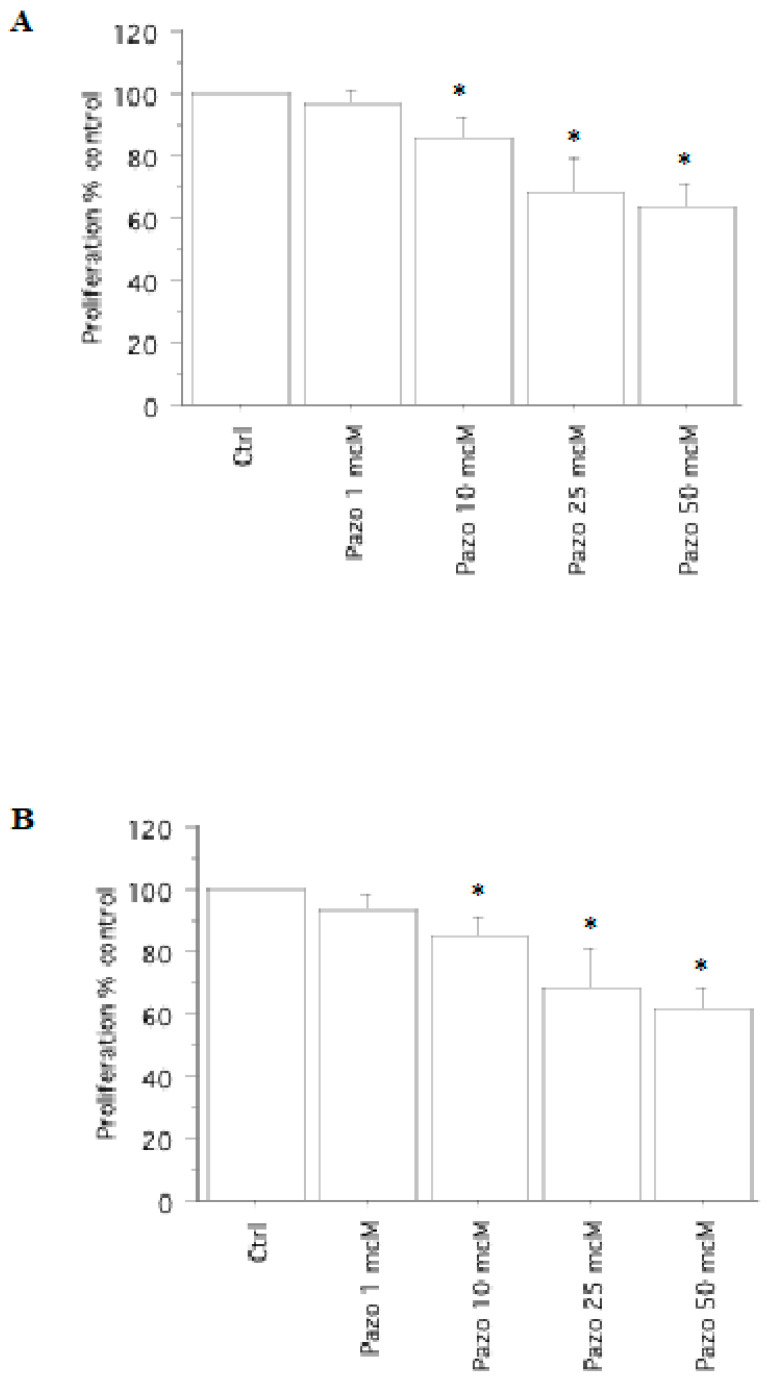
Cell proliferation in pATC cells. WST-1 assay in pATC cells with (**A**) or without (**B**) ^V600E^BRAF mutation, treated with pazopanib (1, 10, 25, or 50 µM) for 24 h. The pATC cell proliferation was significantly reduced vs. control (Ctrl). The obtained results were similar in tumors in presence/absence of the ^V600E^BRAF mutation. Bars represent the mean ± SD. * *p* < 0.05 vs. control according to Bonferroni–Dunn test.

The ^V600E^BRAF mutation was detected in three pATCs; RET/PTC1 and RET/PTC3 were not revealed in pATCs by real-time PCR. The obtained results were similar in tumors in the presence/absence of the ^V600E^BRAF mutation (Figure 2).

### 2.1. Cell Viability and Proliferation Assay

A significant decrease in viability/proliferation (vs. control) was obtained with pazopanib (*p* < 0.01, ANOVA) (Figure 2), in agreement with the results of cell counting.

In pATC, the cell number was 20,104 ± 9850/100 µL, per well, compared to 19,682 ± 995 (98%) with pazopanib 1 µM, 17,490 ± 1100 (87%) with pazopanib 10 µM, 13,872 ± 1115 (69%) with pazopanib 25 µM, and 12,464 ± 1008 (62%) with pazopanib 50 µM (*p* < 0.01, ANOVA). The IC_50_ was 68 µM (through linear interpolation).

### 2.2. Apoptosis

**Figure 3:**
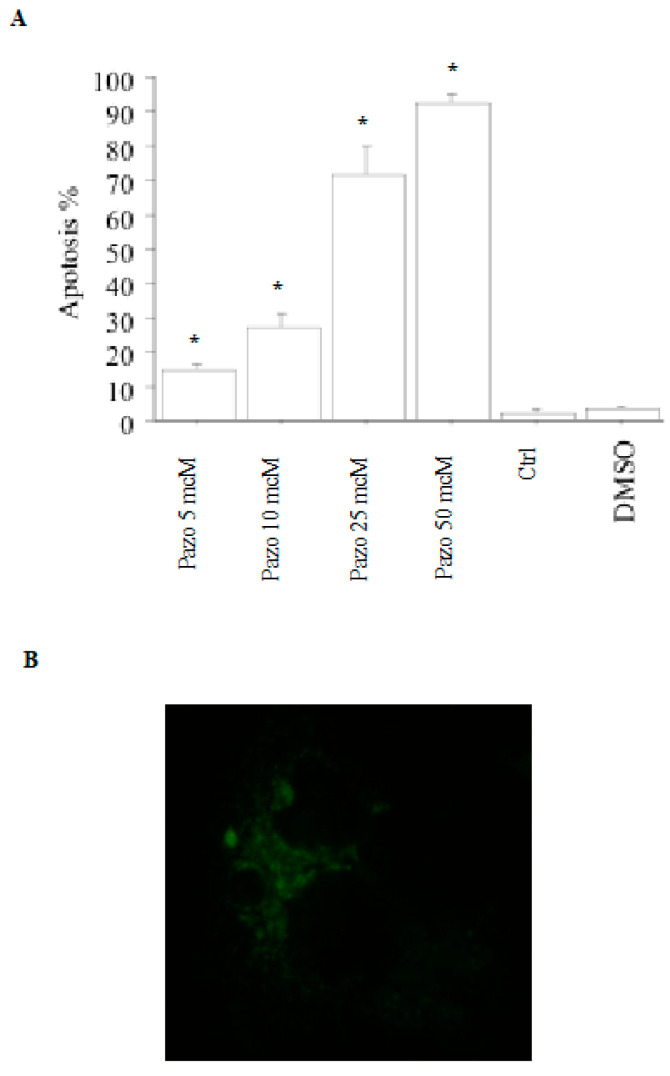
Apoptosis assays in pATC cells. (**A**) pATC cell apoptosis after treatment with pazopanib or with its vehicle (dimethyl sulfoxide) alone for 24 h (by Hoechst 33342). A significant dose-dependent increase in the percentage of apoptotic cells was observed with pazopanib. Data are shown as the mean ± SD, and they were analyzed by one-way ANOVA (with Newman–Keuls multiple comparisons test and a linear trend test) (* *p* < 0.001 vs. control). (**B**) pATC cell apoptosis after treatment with pazopanib 50 µM for 24 h (Annexin V staining).

Upon treating pATC cells with pazopanib (5, 10, 25, or 50 µM), apoptotic cells (expressed as a percentage) increased in a dose-dependent manner. Thus, 14% of the cells were apoptotic upon treating them with pazopanib 5 µM; the higher pazopanib concentrations gave an increased apoptotic rate, up to 28%, 71%, and 91%, respectively (*p* < 0.001, ANOVA; Figure 3A). Annexin V staining confirmed cell apoptosis (Figure 3B).

### 2.3. Migration and Invasion

**Figure 4:**
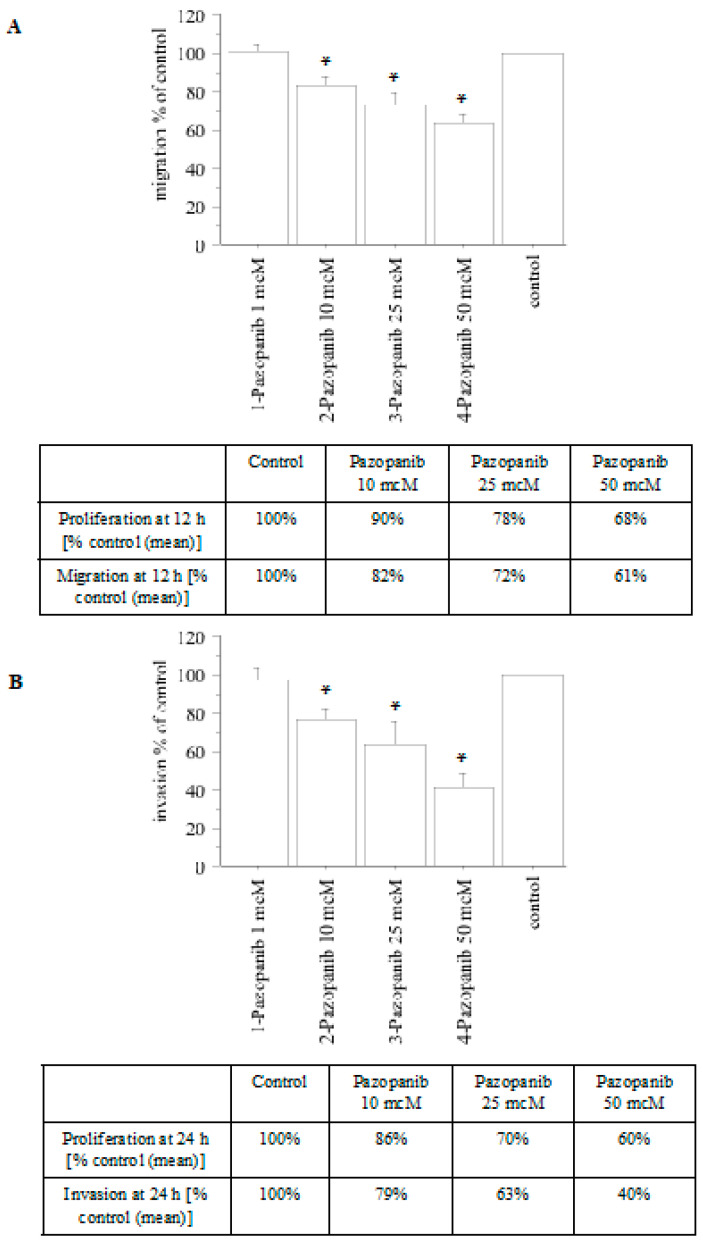
Migration and invasion assays in pATC cells treated with pazopanib. The migration test was performed for 12 h (**A**). For comparison, the inhibition of proliferation at 12 h (as a percentage vs. control) is reported in the table. The invasion test was performed for 24 h (**B**). For comparison, the inhibition of proliferation at 24 h (as a percentage vs. control) is reported in the table. Bars are the mean ± SD. * *p* < 0.05 vs. control (control = medium + FCS 10%) according to Newman–Keuls test.

A decrease in both migration (Figure 4A) and invasion (Figure 4B) was reported upon treating pATC cells with pazopanib.

### 2.4. VEGF Expression

**Figure 5:**
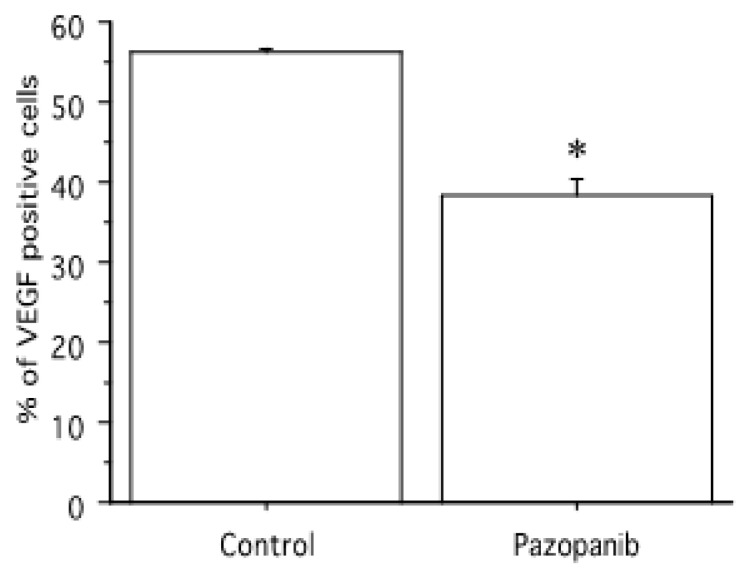
Pazopanib 50 µM significantly decreased VEGF expression in pATC cells. Bars are the mean ± SD. * *p* < 0.05 vs. control.

A decrease in VEGF expression was reported upon treating pATC cells with pazopanib 50 µM (Figure 5).

## 3. Discussion

ATC is one of the most fatal human carcinomas, usually treated by a combination of surgery [5], hyperfractionated accelerated external radiation therapy, and chemotherapy, permitting approximately 6–10 months of median survival [4]. For this reason, the identification of novel treatment strategies is necessary to improve the survival and the quality of life of these patients.

Pazopanib (Votrient) is a multitarget TKI of VEGF receptors, PDGF, and c-Kit, approved by the FDA for the treatment of advanced renal cell carcinoma and advanced soft-tissue sarcoma.

This antiangiogenic drug showed promising clinical activity in DTC patients. A phase 2 trial was conducted in 2009, in 39 patients with RAIR-DTC, metastatic and rapidly progressive, involving the administration of 800 mg daily pazopanib in 4 week cycles until disease progression and/or drug intolerance. The primary endpoint was any tumor response, according to the Response Evaluation Criteria in Solid Tumors 1.0 (RECIST). Confirmed partial responses were observed in 18 patients with a response rate of 49%. It was concluded that pazopanib could represent a promising therapeutic option for these patients [29].

Moreover, the same group of researchers reported a second phase 2 clinical trial of pazopanib in RAIR-DTC, with the aims of evaluating its safety and efficacy in an independent patient cohort, along with the primary endpoint of investigating correlations between early (cycle 1, 4 weeks) changes in Tg level and the RECIST response. Sixty subjects were evaluated, of whom 91.7% were previously treated with systemic therapy beyond RAI. The trial confirmed that pazopanib has clinical activity in these patients [30].

More recently, 168 RAIR-TC patients with progressive disease in the last 12 months were enrolled in the PAZOTHYR study. They were treated with pazopanib for 6 months, and a 35.6% best response rate and 89.4% disease control rate were obtained. Then, 100 patients were randomly assigned (1:1) to be administered with continuous or intermittent pazopanib until progression. Median follow-up of 31.3 months, median time to treatment failure and median progression-free survival were not statistically different between the two groups of patients. The intermittent administration of pazopanib did not have a significantly higher efficacy or tolerance vs. the continuous treatment [31].

The antineoplastic effect of pazopanib has also been evaluated in in vitro studies. After observing the encouraging clinical activity of pazopanib in DTC [29], its preclinical effects were evaluated in ATC, showing that it is able to inhibit the growth of validated ATC cell lines and low-passage patient primary ATC cells at concentrations reached in the plasma of DTC patients who had achieved clinical benefits after the administration of pazopanib monotherapy [29,32]. The combination of pazopanib and microtubule inhibitors (i.e., paclitaxel) exercised a synergistic antineoplastic effect in ATC cells and xenografts, perhaps reflecting an increase in paclitaxel-induced cytotoxicity through cell-cycle-regulatory kinase inhibition by pazopanib [32].

Furthermore, the effects of pazopanib/topotecan combination vs. pazopanib alone were evaluated in the ATC continuous cell lines 8305C and FB3. Proliferation tests were conducted in the ATC cell lines after treatment with pazopanib and/or topotecan for 72 h. Pazopanib and topotecan had a strong synergistic effect in ATC cells. Moreover, a significant decrease in the gene expression of VEGF, hypoxia-inducible factor-1α (HIF-1α), colony stimulating factor-1 (CSF-1), and ATP-binding cassette transporter G2 (ABCG-2) was shown with their combination using real-time PCR [33].

Our study, to the best of our knowledge, is the first to demonstrate the antineoplastic action of pazopanib in primary human ATC cell cultures, showing that it inhibits pATC cell growth in vitro, increases apoptosis, and suppresses the migration and invasion capability. Moreover, pazopanib was able to significantly decrease the VEGF expression in pATC cells.

Thyroid continuous cell lines have been commonly used as preclinical models for research aims [34]. Immortalized cell lines can proliferate without time limitations; although they are easy to handle, they can lose some of their thyroidal intrinsic characteristics by adapting to the in vitro growth conditions [28]. Therefore, these cell lines have some limitations for research on targeted therapies [35]. Primary human cell cultures have some advantages vs. continuous cells in evaluating the antineoplastic effect of different drugs, because primary cells are more comparable in phenotypic features to those that are present in the original tumor. For this reason, they can be used to evaluate the chemosensitivity of cells of each subject in vitro. Although primary cells have a restricted lifespan, they also allow assessing donors, while considering several factors (i.e., age, gender, medical history, race, etc.) [28].

## 4. Materials and Methods

### 4.1. Cell Cultures

#### 4.1.1. Patients Source for Thyroidal Tissue Samples

Surgical thyroidal samples were collected from five subjects with ATC (three females, two males; age range, 55–81; tumor size range, 6–14 cm), at the moment of first surgical operation.

The diagnosis was made on the basis of accepted laboratory, histological, and clinical criteria [36,37,38].

All patients gave their agreement to take part in the study, which received approval from the Ethics Committee of the University of Pisa.

#### 4.1.2. Primary ATC Cell Culture

Firstly, the tissues were finely cut using a scalpel or scissors, and then the fragments were washed 3–5 times in M-199 medium supplemented with 500,000 U/L penicillin, 500,000 U/L streptomycin, and 1,000,000 U/L nystatin (all the mentioned reagents were purchased from Sigma-Aldrich, Merck, Darmstadt, Germany).

Tumor tissue was suspended in DMEM medium (Sigma-Aldrich, Merck) with 10% fetal calf serum (FCS) and maintained at 37 °C in 5% CO_2_.

Once cells reached confluence, they were trypsinized and amplified in tissue culture flasks. Cells were seeded in Methocel at the third passage to assess the colony-forming efficiency, and the biggest ones were amplified in flasks [36,37,38].

Cells were tested for chemosensitivity at the fourth passage (as previously published [22,23]).

#### 4.1.3. Characterization of Thyroidal Samples

The expression of TSH receptor, TPO, Tg, and NIS was evaluated by immunohistochemistry.

Microdissection and DNA extraction, detection of BRAF mutation by PCR single-strand conformation polymorphism (PCR-SSCP), and direct DNA sequencing were performed according to conventional methods previously reported [36,37,38].

### 4.2. Cell Proliferation and Viability Assay

To evaluate proliferation, the proliferation and viability assay using MTT (3-[4,5-dimethylthiazol-2-yl]-2,5-diphenyltetrazolium bromide; Cell Proliferation Reagent WST-1, Sigma-Aldrich, Merck) was used [36,38,39]. The tetrazolium salts are cleaved to formazan by cellular mitochondrial dehydrogenases. An expansion in the number of viable cells results in an increase in the overall activity of these enzymes. This increase in enzyme activity leads to an increment in the amount of formazan dye formed, which directly correlates to the number of metabolically active cells in culture. The formazan dye produced by metabolically active cells was quantified by a scanning multi-well spectrophotometer (ELISA reader) by measuring the absorbance of the dye solution at 450 nm.

Cells were plated in a 96-well microtiter plate at a concentration of 35,000 cells/mL in a final volume of 100 µL per well. Then, various concentrations of pazopanib (1, 10, 25, and 50 µM) or its vehicle (dimethyl sulfoxide (DMSO)) alone were added to the wells in quadruplicate, for 24 h. After the treatment period, 10 µL of the Cell Proliferation Reagent WST-1 was added to 100 µL of culture medium in each well, and the absorbance of the samples was measured at 450 nm against the control (the same cells without any treatment). The same volume of culture medium and WST-1 (10 µL of Cell Proliferation Reagent WST-1/100 µL culture medium) was added to one well, as a background control (absorbance of culture medium plus WST-1 in the absence of cells).

Then, 2 h after the beginning of the tetrazolium reaction, the absorbance was measured at 450 nm. The determination of the IC_50_ was achieved by linear interpolation.

The absorbance of blank was subtracted from the one of the control and treatments. The control was normalized to 100% for each assay, and treatments were expressed as a percentage of the control.

### 4.3. Cell Counting

The proliferation was also assessed by cell number counting [36,38,39].

Since the MTT assay measures mitochondrial cell activity, and since it has already been demonstrated that there is not always a direct relationship with cell number, the proliferation was also evaluated using cell number counting. Cells were seeded at a density of 13,000 cells per well in 24-well tissue culture plates in medium supplemented with 10% FCS with or without the indicated factors. The medium was changed every other day. After 72 h in an atmosphere of 5% CO_2_ at 37 °C, cells were detached from plates by incubation with 500 mL of PBS containing 100 mg of trypsin and 1 mM EDTA. Cells were counted using a hemocytometer.

The concentrations of pazopanib required for 50% inhibition of growth (IC_50_) were calculated by linear regression analysis of the obtained dose–response curves.

### 4.4. Apoptosis Determination

#### 4.4.1. Hoechst Uptake

pATC cells were plated (35,000 ATC cells/mL in 100 µL per well), and then treated with pazopanib (5, 10, 25, or 50 µM) for 24 h (at 37 °C, 5% CO_2_). Next, cells were stained with Hoechst 33342 [39]. The apoptosis index (apoptotic/total cells ratio × 100) was calculated.

#### 4.4.2. Annexin V Binding Test

Cells were seeded in a Lab-TekII Chamber Slide System (Nalge Nunc International, ThermoFisher Scientific, Waltham, MA, USA), treated with pazopanib for 24 h, and then tested as previously reported [39].

### 4.5. Migration and Invasion

Cell migration and invasion were performed in 96-well Transwell Permeable Supports (Corning Life Sciences, Sigma-Aldrich, Merck) according to the manufacturer’s instructions [23,40]. In short, cells were starved in serum-free medium at 5% CO_2_ and 37 °C for 5 h, using a PBS solution with 5 mM EDTA. The total cell number was estimated. After centrifugation, cells were plated (0.5 × 10^5^ cells/well) in serum-free medium.

The migration test was performed for 12h. For the invasion assay, the inserts were coated overnight with a basement membrane extract (Trevigen, Bio-Techne, Minneapolis, MN, USA) solution (37 °C, 5% CO_2_) before plating cells. Then, FCS 10% vol/vol or serum-free medium was put into receiver wells; where required, increasing concentrations of pazopanib were added to both Transwell chambers for 24 h. After removing the medium, a solution of calcein AM (2 μg/mL; Sigma-Aldrich, Merck) was added to the lower compartments for 1 h. Intracellular fluorescence was determined using a 96-well plate reader (at 485 nm for excitation and 520 nm for emission). For each assay, a standard curve was established to convert the fluorescence values to the number of migrated or invasive cells.

### 4.6. Immunocytochemistry for VEGF Expression

VEGF expression was conducted with an anti-VEGF rabbit polyclonal antibody (Santa Cruz Biotechnology) at a 1:50 dilution and reported as a percentage (%) of positive cells with the respect to a total of at least 1000 tumor cells [40].

### 4.7. Statistics

For normally distributed variables, data were shown as the mean ± standard deviation (SD), or as the median and interquartile range. The experiments were conducted thrice with the primary cells established from each subject (the mean of five samples is reported). One-way ANOVA was used to compare the mean group values for normally distributed variables; alternatively, the Mann–Whitney U or Kruskal–Wallis test was used. The χ^2^ test was applied to compare proportions. The Bonferroni–Dunn test was used for post hoc comparisons on normally distributed variables. Data regarding apoptosis were evaluated by one-way ANOVA with the Newman–Keuls multiple comparisons test.

## 5. Conclusions

In conclusion, we show for the first time the antineoplastic action of pazopanib in primary human ATC cell cultures, demonstrating in vitro that it is able to inhibit primary ATC cell proliferation, increase apoptosis, and suppress the migration and invasion capability. Moreover, pazopanib was able to significantly decrease the VEGF expression in pATC cells.

Personalized medicine, which considers the specific features of the disease in each patient, represents the future for a more precise treatment approach in each patient, and primary human cell cultures can be a good model to evaluate the antineoplastic effect of various drugs.

## Data Availability

The data presented in this study are available within the article.
